# MicroRNA-Based Therapeutics for Drug-Resistant Colorectal Cancer

**DOI:** 10.3390/ph14020136

**Published:** 2021-02-08

**Authors:** Eunsun Jung, Jinhyeon Choi, Jang-Seong Kim, Tae-Su Han

**Affiliations:** Korea Research Institute of Bioscience and Biotechnology (KRIBB), Daejeon 34141, Korea; silverline09@kribb.re.kr (E.J.); cluke@kribb.re.kr (J.C.)

**Keywords:** microRNA, chemoresistance, targeted therapy, immunotherapy, anti-cancer drug resistance, colorectal cancer

## Abstract

Although therapeutic approaches for patients with colorectal cancer (CRC) have improved in the past decades, the problem of drug resistance still persists and acts as a major obstacle for effective therapy. Many studies have shown that drug resistance is related to reduced drug uptake, modification of drug targets, and/or transformation of cell cycle checkpoints. A growing body of evidence indicates that several microRNAs (miRNAs) may contribute to the drug resistance to chemotherapy, targeted therapy, and immunotherapy by regulating the drug resistance-related target genes in CRC. These drug resistance-related miRNAs may be used as promising biomarkers for predicting drug response or as potential therapeutic targets for treating patients with CRC. In this review, we summarized the recent discoveries regarding anti-cancer drug-related miRNAs and their molecular mechanisms in CRC. Furthermore, we discussed the challenges associated with the clinical application of miRNAs as biomarkers for the diagnosis of drug-resistant patients and as therapeutic targets for CRC treatment.

## 1. Introduction

Colorectal cancer (CRC) occurs in both men and women, and there were 1.8 million cases worldwide in 2018, that resulted in approximately 880,800 deaths [1,2,3]. CRC initially develops as two mucosal colonic polyps, including hyperplastic and adenomatous, and histological and molecular evidence indicates that most CRCs arise from adenomas. The adenoma gradually transforms into an advanced adenocarcinoma, which becomes an invasive carcinoma and eventually metastasizes [4,5]. CRC stages are categorized into four phases (stages I–IV) according to the American Joint Committee on Cancer tumor, node, metastasis staging guidelines [1,3]. Cancer prevention and early diagnosis with polypectomy can reduce CRC mortality. Despite the use of various screening methods to identify CRC, many patients still suffer from treatment failure and recurrence [3,5,6,7]. Therefore, an effective strategy to improve patient survival is development of novel diagnostic markers for early detection and potent therapeutic strategies.

Multiple genetic events, including mutations and epigenetic modifications have been identified during CRC development, such as Wnt, RAS, and p53 signaling pathways. Moreover, the molecular alterations in these pathways are associated with CRC progression. The most representative mutation is the Wnt pathway-related adenomatous polyposis coli (*APC*) gene. *APC* prevents the accumulation of the transcription factor β-catenin; when *APC* is mutated, the transcription of proto-oncogenes is activated, leading to the formation of multiple polyps [8,9]. Subsequently, activating mutations in *KRAS*, which plays a major role in tumorigenesis through the MAPK and PI3K pathways, occur in the early to intermediate stages of adenoma [3,5]. In contrast, *SMAD4* loss-of-function mutations occur in the intermediate to late stages of adenoma and mutations in *TP53*, a well-known tumor suppressor, leading to the development of late adenoma to adenocarcinoma [3,4].

Typically, the ideal CRC treatment is surgical resection to completely remove the tumor mass. However, this method has several limitations because a quarter of patients with CRC are diagnosed at an advanced stage with distant metastasis, resulting in difficulties in surgical resection and subsequent reduced overall survival. For those patients, the treatment goal is shrinkage of the tumor and blocking further tumor growth. Therefore, radiotherapy, chemotherapy, targeted therapy, or immunotherapy are leading strategies for controlling advanced stage tumors in patients with CRC. In some cases, chemo- or radiotherapy can be applied with surgery as adjuvant or neoadjuvant treatment. Chemotherapeutic drugs such as 5-fluorouracil (5-FU), capecitabine, and oxaliplatin inhibit DNA synthesis via various mechanisms and lead to cell death [10,11].

The concept of molecular targeted therapy was first proposed in the early 1900s and applied to cancer treatment in 1988 [12]. Targeted therapies directly inhibit cancer cell growth and motility. Most targeted therapies consist of monoclonal antibodies that can inactivate specific enzymes, thereby blocking cell growth and inducing apoptosis. The first targeted therapeutic agent for CRC was cetuximab in 2004. Subsequently, numerous targeted agents have been developed, such as anti-vascular endothelial growth factor (VEGF)/VEGF receptor (VEGFR) (bevacizumab, ziv-aflibercept, regorafenib, and ramucirumab), anti-epidermal growth factor receptor (EGFR) (cetuximab and panitumumab), and anti-BRAF agents (vemurafenib and dabrafenib). In addition, to enhance immune surveillance and suppression against cancer cells, immune checkpoint therapy has been developed. Many cancer cells are known to evade T-cell detection. Immunotherapy creates an environment that allows individual immune systems to better recognize cancer cells. The targeting of immune checkpoint inhibitors, which are typically turned on/off in cancer, restores the immune response toward cancer cells. For example, programmed cell death protein 1 (PD-1) is a T-cell protein that helps prevent attack on cancer cells; hence, the use of PD-1 inhibitors improves the immune response to cancer. In CRC treatment, novel T-cell bispecific antibodies targeting the carcinoembryonic antigen of tumor cells and CD3 of T cells are used for targeting the cancer cells. These immune checkpoint blockade agents have shown potent antitumor activity by increasing intra-tumoral T-cell penetration and by activating and upregulating PD-1/PD-L1 expression in a preclinical model [13,14,15]. However, despite these medical advances, chemotherapy, targeted therapy, and immunotherapy have shown a high rate of failure. This may be caused by invasion and metastasis of cancer-related drug resistance, resulting in decreased patient survival.

**Figure 1:**
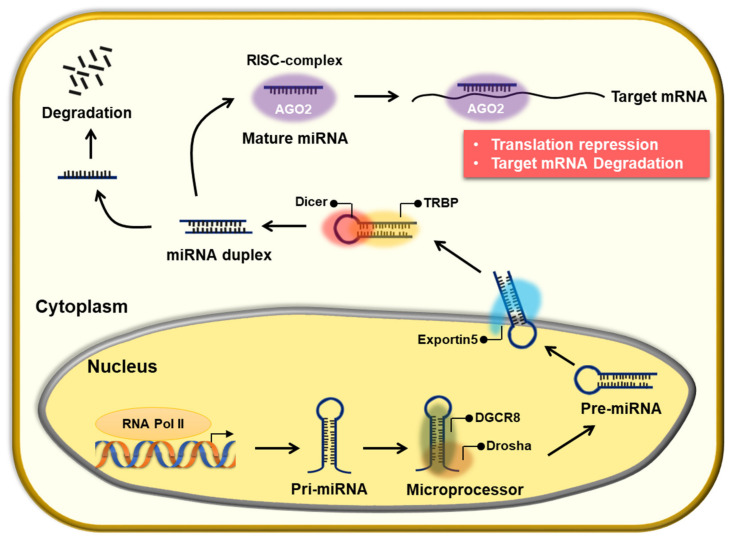
The canonical pathway of microRNA (miRNA) processing. In the nucleus, miRNA biogenesis is initiated with RNA polymerase II. Pri-miRNA with a hairpin transcript is further processed to pre-miRNA by the DGCR8/Drosha microprocessor complex. The pre-miRNA is then translocated to the cytoplasm through exportin-5. In the cytoplasm, multidomain human TAR element-binding protein (TRBP) recognizes pre-miRNA and is cleaved by Dicer, resulting in miRNA maturation. In the miRNA-duplex, only one strand is loaded onto the RNA-induced silencing complex (RISC) complex (including the AGO1-4 protein), which then binds to the complementary target gene. Finally, the target mRNA can be suppressed through translation repression or degradation.

MicroRNAs (miRNAs) are small non-coding RNAs that were first discovered in *Caenorhabditis elegans*. They are involved in most biological processes, including development, differentiation, proliferation, growth, and apoptosis, as they finely regulate the expression of target genes [2,16,17]. Canonical miRNA biogenesis consists of three major processes. First, miRNAs are processed into primary miRNAs (pri-miRNAs) with hairpin structures by RNA polymerase II (Pol II) in the nucleus, which are then converted to precursor miRNAs (pre-miRNAs) with a stem-loop structure by the RNase III, Drosha. Subsequently, Drosha forms a dimer with DGCR8/Pasha, a dsRNA-binding protein, and functions as a microprocessor. When the pre-miRNA is transported from the nucleus to the cytoplasm, it is processed into a 20–25 bp-long miRNA-miRNA duplex by the RNase III, Dicer. Only one strand from the duplex regulates target gene expression by directly binding to the complementary mRNA sequences of target genes within the RNA-induced silencing complex (RISC; Figure 1) [18,19]. MiRNAs target many genes; however, their regulation is perturbed in cancers or other diseases. Depending on the target genes in cancer, miRNAs are referred to as oncogenic miRNAs (oncomiRs) or tumor suppressor miRNAs [20,21]. For example, the miR-17-92 cluster, called oncomiR, is overexpressed in various cancer types owing to abnormal amplification of *Myc* [22]. In contrast, let-7, a representative miRNA that acts as a tumor suppressor, is poorly expressed in malignant tumors. In addition, various miRNAs have been studied in other cancers [23].

Recent reports show that these miRNAs are associated with anti-cancer drug resistance by regulating cell growth, apoptosis, hypoxia, angiogenesis, and epithelial-mesenchymal transition (EMT). For instance, (1) miR-125b and miR-504 reduce apoptosis by inhibiting the expression of p53 [24,25]; (2) miR-34a reduces apoptosis by inhibiting the expression of SIRT1, which regulates p53 [24,26]; (3) miR-138 expression is reduced in cancer; however, the expression of the target gene, *TERT,* is increased, resulting in infinite proliferation [27]; (4) expression of miRNAs such as miR-15b, miR-16, miR-20a, and miR-20b decreases hypoxia, increasing the expression of angiogenic factors such as VEGF, which enables angiogenesis; (5) miR-200 and miR-205 target the E-cadherin transcription inhibitors ZEB1 and ZEB2 that are involved in EMT, thereby preventing transcriptional inhibition and eventually increasing EMT, a characteristic of cancer metastasis [26].

In this review, we summarized the current knowledge on drug resistance-related miRNAs and their target genes with molecular mechanisms of acquired drug resistance and proposed novel strategies to overcome anti-cancer drug resistance.

## 2. The Role of miRNAs in Chemoresistance

Chemotherapy is an important strategy for treating cancer, although its effectiveness is limited by drug resistance. Therefore, chemoresistance of cancer cells has to be overcome using novel strategies, and for this, understanding its underlying molecular mechanisms is of paramount importance. According to previous studies, chemoresistance is known to be associated with oncogenes, tumor suppressor genes, DNA repair, EMT, and cancer stemness, all of which can be controlled by miRNAs. Previously, only 5-FU was used for standard chemotherapy. However, currently, various drugs, including irinotecan, oxaliplatin, and capecitabine, are being used in combination therapy; nonetheless, chemoresistance remains a major problem. We discuss the roles of miRNAs in chemoresistance and sensitivity below and summarized in Table 1.

### 2.1. The Role of miRNA in 5-FU-Resistant CRC

5-FU and its prodrug capecitabine are used as the standard prescription for patients with CRC. 5-FU is a nucleobase analogue of uracil, which inhibits tumor growth by blocking DNA synthesis. Several studies have shown that miRNAs can affect 5-FU resistance and sensitivity by regulating Wnt/β-catenin, EGFR, transforming growth factor (TGF)-β, and other signaling pathways.

The Wnt/β-catenin signaling pathway plays an important role in the initiation and progression of CRC. Mutations in β-catenin, axin, GSK3β, and *APC* lead to aberrant signaling and development of cancer. Yu et al. have shown that miR-125b regulates the CXCL12/CXCR4 axis by suppressing *APC* expression. An in vivo study has shown that miR-125b induces 5-FU resistance in a xenograft model [28]. In contrast, miR-149 and miR-320 increase 5-FU sensitivity by decreasing forkhead box protein M1 (FOXM1) expression, which subsequently promotes β-catenin localization in the nucleus [29,30].

Mutations in the PI3K/AKT pathway are common in CRC development and 5-FU resistance. Reports have shown that miRNAs are involved in 5-FU resistance via the PI3K/AKT signaling pathway. MiR-135b and miR-182 promote 5-FU resistance of CRC by downregulating ST6GALNAC2 and activating the PI3K/AKT signaling pathway [31]. MiR-204 upregulates 5-FU sensitivity via the PI3K/AKT signaling pathway; it inhibits HMGA2 expression [32], thereby decreasing PI3K/AKT signaling. In contrast, miR-587 activates PI3K/AKT signaling and directly inhibits PPP2R1B expression, inducing 5-FU resistance [33]. MiR-200c also induces chemoresistance by directly inhibiting PTEN, the negative regulator of the PI3K/AKT pathway, and activating caspase-3 [34]. Insulin-like growth factor type 1 receptor (IGF-1R) contributes to the development and progression of CRC by activating the PI3K/AKT pathway. MiR-302a inhibits IGF-1R and downregulates the AKT signaling pathway [35]. Liu et al. have shown that miR-302a enhances 5-FU sensitivity and promotes cell death.

Upstream regulators of PI3K are also known to be involved in regulating 5-FU resistance. MiR-224 knockdown increases 5-FU chemosensitivity by creating *KRAS* ‘mutant-like’ expression [36]. Another upstream regulator, EGFR, is controlled by miR-20b. MiR-20b inhibits ADAM9 expression and activates the EGFR-AKT pathway. Thus, miR-20b decreases 5-FU resistance by downregulating the ADMA9/EGFR/AKT pathway in CRC [37].

The canonical TGF-β signaling pathway involving Smad2/3 inhibits proliferation and promotes apoptosis of abnormal cells, whereas mutations in the TGF-β signaling pathway promote proliferation and inhibit apoptosis. Mutations in the TGF-β signaling pathway are commonly found in CRC, indicating that regulation of this pathway is critical for carcinogenesis. *Smad2* is regulated by miR-552. Overexpression of miR-552 significantly downregulates 5-FU resistance in CRC [38], whereas, miR-106a promotes 5-FU resistance. MiR-106a inhibits TGFβR2 and metastasis [39].

Mutation in the *TP53* signaling pathway is a major regulator of CRC progression. The *TP53* mutation has been detected in most CRC cases, where it performs an oncogenic function. Han et al. have shown that the miR-338-3p/mTOR/P70s6k axis induces 5-FU resistance. The miR-338-3p inhibitor activates mTOR and sensitizes *p53*-mutant and -deficient cells [40]. In contrast, miR-339 increases 5-FU sensitivity in CRC. MiR-339 negatively regulates MDM2 expression. Downregulation of MDM2 increases TP53 protein expression and activates stress response during 5-FU treatment [41].

Other signal transductions are also involved in 5-FU resistance of CRC, such as the nuclear factor (NF)-κB and apoptosis-related signaling pathways. NF-κB is known to play an important role in inflammation and cell survival. MiR-557 inhibits heat shock protein 27 (HSP27) expression and promotes 5-FU sensitivity [42]. MiR-15b also promotes 5-FU sensitivity. Overexpression of miR-15b downregulates NF-κB and IκB kinase α and induces apoptosis in vitro and in vivo [43].

### 2.2. The Role of miRNA in Oxaliplatin-Resistant CRC

Oxaliplatin, a platinum-based chemical containing 1,2-diaminocyclohexane (DACH), binds to the guanines and cytosines of DNA and leads to cross-linking of DNA. Cross-linked oxaliplatin inhibits DNA synthesis and transcription. High concentrations of this drug can also suppress RNA synthesis. The structure of oxaliplatin is similar to that of cisplatin, although it has fewer side effects than cisplatin [44]. For CRC treatment, oxaliplatin is typically used with 5-FU and leucovorin, i.e., a formulation called Folfox.

MiR-103/107 promotes the Wnt/β-catenin signaling pathway by directly inhibiting Axin2 expression. Elevated Wnt/β-catenin signaling contributes to chemoresistance and tumor recurrence in xenograft models of CRC [45]. MiR-506 increases oxaliplatin sensitivity by inhibiting *MDR1*/P-gp expression in CRC. Zhou et al. have shown that overexpression of miR-506 in HCT116 cells promotes oxaliplatin sensitivity by suppressing the Wnt/β-catenin signaling pathway [46].

Mutations in the PI3K/AKT pathway are also related to oxaliplatin resistance. MiR-17 regulates the expression of PTEN and contributes to chemoresistance and sensitivity to oxaliplatin, irinotecan, and 5-FU [47]. MiR-19a also regulates PTEN expression but reverses the chemoresistance of oxaliplatin via the PTEN/PI3K/AKT pathway [48]. MiR-224 upregulates sensitivity to oxaliplatin by inhibiting HMGA2 as well as RAB22A [32,49]. MiR-325 downregulates HSPA12B expression and increases oxaliplatin sensitivity via the HSPA12B/PI3K/AKT/Bcl-2 axis [50]. Upstream of the PI3K/AKT pathway, IGF-1R is inhibited by miR-143, inactivating the AKT signaling pathway and downregulating HIF-1α. Overexpression of miR-143 inhibits tumor growth and increases chemosensitivity to oxaliplatin [51].

Several miRNAs that are associated with the TGF-β signaling pathway induce oxaliplatin chemoresistance. MiR-34a downregulates *Smad4* expression and inhibits TGF-β signaling. Downregulation of TGF-β signaling increases oxaliplatin sensitivity by activating macroautophagy [52]. MiR-19b-3p also inhibits Smad4 expression and increases oxaliplatin sensitivity [53]. Ye et al. have shown that miR-4666-3p and miR-329 synergistically suppress TGF-β signaling and increase oxaliplatin sensitivity [54]. MiR-4666-3p inhibits TGF-βR1, and miR-329 inhibits TGF-β1. The suppression caused by these two miRNAs effectively suppresses TGF-β signaling and increases oxaliplatin sensitivity.

Among other signaling pathways, miR-625-3p increases oxaliplatin resistance by inhibiting MAP2K6 expression. Inhibited MAP2K6 causes non-canonical signaling of p38 MAPK [55]. MiR-122 inhibits the X-linked inhibitor of the apoptosis protein. Overexpressed miR-122 induces oxaliplatin sensitivity by inhibiting apoptosis [56].

The apoptosis-related signaling pathway is closely related to oxaliplatin resistance. MiR-503 directly inhibits PUMA expression and its overexpression increases chemoresistance to oxaliplatin in p53-null cells. Xu et al. shown that miR-503 may be involved in the progression of multidrug-resistance (MDR) [57]. MiR-218 enhances the chemosensitivity of oxaliplatin by suppressing YEATS4 expression and inducing apoptosis [58].

### 2.3. The Role of miRNA in Irinotecan-Resistant CRC

Irinotecan (CPT-11) is a derivative of camptothecin that inhibits topoisomerase-I, an enzyme related to DNA replication. Irinotecan is a key drug for treating CRC. Treatment with the combination of irinotecan and 5-FU or oxaliplatin prolongs survival more than treatment with 5-FU or oxaliplatin alone [59]. As mentioned previously, mutations in the PI3K/AKT pathway are related to chemoresistance. MiR-17 regulates PTEN expression and contributes to the sensitivity to oxaliplatin, irinotecan and 5-FU [47]. Chang et al. demonstrated that VAPA, which acts downstream of AKT phosphorylation, is a target of miR-194. Overexpression of miR-194 increases the sensitivity of HCT116 cells to oxaliplatin and irinotecan [60]. Targeting of TGF-β is also a good strategy for overcoming irinotecan resistance. Overexpression of miR-146a increases TGF-β and interleukin-10 expression and chemosensitivity of HT29 cells to 5-FU or irinotecan [61]. ABCG2 is a known MDR transporter that utilizes 5-FU and irinotecan as its substrates. MiR-519c inhibits ABCG2 and HuR expression, the latter of which is an activator protein of ABCG2. Overall, overexpression of miR-519c increases sensitivity to irinotecan [62]. MiR-514b-3p suppresses EMT by inhibiting *FZD4* and *NTN1*. Overexpression of miR-514b-3p increases sensitivity to cisplatin and irinotecan [63].

**Table 1:** MicroRNAs (miRNAs) involved in chemoresistance in colorectal cancer (CRC).

miRNA	Drug	Target Gene	Signaling Pathway	Level	Reference
miR-125b	5-FU	APC	Wnt/β-catenin	Up	[28]
miR149	5-FU	FOXM1	Wnt/β-catenin	Down	[29]
miR320	5-FU	FOXM1	Wnt/β-catenin	Down	[29]
miR-135b	5-FU	ST6GALNAC2	PI3K/AKT	Up	[31]
miR-182	5-FU	ST6GALNAC3	PI3K/AKT	Up	[31]
miR-204	5-FU	HMGA2	PI3K/AKT	Down	[32]
miR-587	5-FU	PPP2R1B	PI3K/AKT	Up	[33]
miR-200c	5-FU	PTEN	PI3K/AKT	Up	[34]
miR-302a	5-FU	IGF-1R	PI3K/AKT	Down	[35]
miR-224	5-FU	KRAS	PI3K/AKT	Down	[36]
miR-20b	5-FU	ADAM9	PI3K/AKT	Down	[37]
miR-552	5-FU	Smad2	TGF-β	Down	[38]
miR-106a	5-FU	TGF-βR2	TGF-β	Up	[39]
miR-338-3p	5-FU	mTOR	p53	Up	[40]
miR-339	5-FU	MDM2	p53	Down	[41]
miR-557	5-FU	HSP27	Other	Down	[42]
miR-15b	5-FU	NF-𝜅B, IKKα	Other	Down	[43]
miR-103	oxaliplatin	Axin2	Wnt/β-catenin	Up	[45]
miR-107	oxaliplatin	Axin2	Wnt/β-catenin	Up	[45]
miR-506	oxaliplatin	MDR1/P-gp	Wnt/β-catenin	Down	[46]
miR-17	5-FU, oxaliplatin, irinotecan	PTEN	PI3K/AKT	Down	[47]
miR-19a	oxaliplatin	PTEN	PI3K/AKT	Up	[48]
miR-204	oxaliplatin	HMGA2, RAB22A	PI3K/AKT	Down	[32,49]
miR-325	oxaliplatin	HSPA12B	PI3K/AKT	Down	[50]
miR-143	oxaliplatin	HIF-1α	PI3K/AKT	Down	[51]
miR-34a	oxaliplatin	Smad4	TGF-β	Down	[52]
miR-19b-3p	oxaliplatin	Smad4	TGF-β	Down	[53]
miR-4666-3p	oxaliplatin	TGF-βR1	TGF-β	Down	[54]
miR-329	oxaliplatin	TGF-β1	TGF-β	Down	[54]
miR-625-3p	oxaliplatin	MAP2K6	Other	Up	[55]
miR-122	oxaliplatin	XIAP	Other	Down	[56]
miR-503	oxaliplatin	PUMA	Other	Up	[57]
miR-218	oxaliplatin	YEATS4	Other	Down	[58]
miR-194	oxaliplatin, irinotecan	VAPA	PI3K/AKT	Down	[60]
miR-146a	5-FU, irinotecan	TGF-β, IL-10	TGF-β	Up	[61]
miR-519c	irinotecan	ABCG2, HuR	Other	Down	[62]
miR-514-3p	irinotecan	FZD4, NTN1	Other	Down	[63]

## 3. Molecular Targeted Therapeutic Drugs and Resistance

### 3.1. VEGF/VEGFR Targeted Therapy

Angiogenesis is essential for the growth, invasion, and metastasis of many solid tumors. VEGF is a representative pro-angiogenic factor. A target drug that blocks the VEGF/VEGFR pathway is being tested clinically for treating CRC [64]. VEGF inhibitors used in CRC include bevacizumab (Avastin), regorafenib (Stivarga), and aflibercept (Zaltrap). Bevacizumab is a U.S. Food and Drug Administration-approved monoclonal antibody targeting VEGF-A, and is being used for treating metastatic colon cancer alone or in combination with chemotherapeutic drugs, including 5-FU [64,65,66]. Bevacizumab exerts a good curative effect on most patients with metastatic CRC, although some patients do not respond to it or develop chemoresistance. Recently, mutations in PTPRT, a phosphatase involved in JAK/STAT signaling, have been detected in patients with bevacizumab resistance, and reports show that resistance to bevacizumab increases when FOXF1 is overexpressed [67,68].

Regorafenib (Stivarga) is a multi-kinase inhibitor that inhibits the VEGFR-TIE2 tyrosine kinase, VEGFR-1,2,3, platelet-derived growth factor, and fibroblast growth factor. It is used for treating metastatic CRC, advanced gastric cancer, and advanced hepatocellular carcinoma and has been reported to increase the overall survival of patients with metastatic CRC [69,70]. However, its use is limited owing to the development of resistance. Several studies have shown that *Notch-1* is significantly upregulated in resistant cells by 1 µM of regorafenib, while sensitivity to regorafenib is restored when *Notch-1* is inhibited [71]. In addition, a recent study has reported that CCR2, a CC chemokine receptor 2, is upregulated in regorafenib-resistant cells and that the positive feedback loop of CCR2/β-catenin sustains resistance [72].

Aflibercept (Zaltrap) is a VEGF-A/VEGF-B inhibitor that binds to VEGF and acts as a “VEGF trap,” inhibiting the growth of new blood vessels in metastatic CRC [73]. It inhibits the migration and invasion of drug-resistant CRC, thereby exhibiting therapeutic effects [74,75].

### 3.2. EGFR-Targeted Therapy

EGFR, also known as ErbB1 or HER1, activates cell growth-related pathways when EGF binds to the receptor domain. EGFR is highly expressed and mutated in cancer cells, and this activates downstream signaling in many cancer cells [76,77]. EGFR inhibitors inhibit cancer cell growth by binding to a specific part of EGFR and blocking signaling. EGFR inhibitors include tyrosine kinase inhibitors, which bind to the tyrosine kinase domain and halt its activity, as well as monoclonal antibodies that prevent the binding of growth factors to the extracellular components of EGFR [78].

EGFR inhibitors used for treating CRC include cetuximab (Erbitux) and panitumumab (Vectibix). Cetuximab is a monoclonal antibody that binds to EGFR and inhibits EGFR signaling. It is used for treating metastatic CRC harboring wild type *KRAS*, which encodes a small G protein involved in the EGFR pathway [65,79]. A therapeutic effect is not observed if *KRAS* is mutated; hence, genetic mutation screening should be conducted before the treatment. Acquired resistance to cetuximab has been reported in patients with metastatic CRC. It is remarkable that *HER2* amplification has been detected in the circulating tumor DNA of patients who acquired cetuximab resistance, and *HER2* amplification before/after cetuximab treatment differs significantly [80]. According to the results of a recent study that analyzed the exosomes of cetuximab-resistant patients, the expression of UCA1, an exosomal long non-coding RNA (lncRNA), is remarkably high, and can predict the clinical outcome of cetuximab treatment in patients with CRC [81].

Panitumumab (Vectibix) is a humanized monoclonal antibody targeting EGFR. It binds to the extracellular domain of EGFR and blocks the cascade of intracellular signals. Similar to cetuximab, it targets EGFR and shows similar activities, although the mechanism of action varies slightly. Similar to cetuximab, it is not applied to patients with *KRAS* or *NRAS* mutations, as the treatment efficacy is insufficient in such patients [82,83,84]. Owing to the resistance to panitumumab, several studies have screened biomarkers related to the resistance in patients with metastatic colon cancer who have acquired panitumumab resistance [77]. Using Nanostring nCounter-based analysis, researchers have confirmed the expression signatures of *ERBB2*, *MLPH*, *IRX3*, *MYRF*, and *KLK6* in patients with panitumumab resistance [83,84]. In addition, EGFR inhibitors such as erotinib, naratinib, and osimertinib target the tyrosine kinase domain, and the drugs mentioned above are the ones that are mainly used for treating CRC.

### 3.3. BRAF-Targeted Therapy

Vemurafenib is a BRAF inhibitor that interrupts BRAF/MEK signaling in the BRAF/MEK/ERK pathway and was first developed for the treatment of melanoma. The therapeutic effect is observed only in the presence of a mutation in which valine at position 600 of the BRAF protein is converted to glutamate [85,86,87]. Clinical trials have also reported this drug to affect CRC. A school of thought posits that the persisting cancer stem cells (CSCs) are the main causes of the acquisition of drug resistance in CRC [88]. According to a recent study, the expression of ErbB-3 in CSC increases significantly with the development of vemurafenib resistance, which is alleviated when ErbB-3 is knocked down [89,90,91].

Dabrafenib and encorafenib are also targeted therapeutic drugs that target mutated *BRAF*. These mutated *BRAF* inhibitors can be used in combination with EGFR inhibitors to increase therapeutic effects in clinical trials. The results of clinical trials show that dabrafenib exerts a therapeutic effect in patients with CRC who harbor mutated V600E *BRAF* with the EGFR inhibitor panitumumab as combination treatment [82]. Similarly, encorafenib is also clinically effective when used in combination with cetuximab, an EGFR inhibitor [92].

### 3.4. Roles of miRNAs in Targeted Therapy Resistance

Using the aforementioned approach, many cancer patients have benefited from molecular targeted therapy. However, the occurrence of drug resistance often leads to poor prognosis. In this section, we describe how to modulate drug resistance using miRNA and its target genes (Table 2).

The lncRNA MIR100HG is a polycistronic miRNA encoding miR-100 and miR-125b. In the case of cetuximab-resistant CRC, the expression levels of MIR100HG, miR-100, and miR-125b are concomitantly upregulated. These two miRNAs regulate Wnt signaling by reducing the expression of five negative regulators of Wnt signaling. Researchers argue that inhibition of MIR100HG and Wnt signaling may help overcome cetuximab resistance [93]. According to a recent report, microarray assays were performed on CRC cell lines and patient tissues resistant to cetuximab, results of which confirmed that miR-302a expression was significantly reduced. The expression of NFIB and CD44, which miR-302a targets, decreased. Metastasis was inhibited, and cetuximab sensitivity increased when miR-302a expression increased in cetuximab-resistant CRC cells [94]. Similarly, in another study, miRNA chips were analyzed to identify miRNAs that contribute to cetuximab resistance in CRC. Among the candidate miRNAs, miR-199-5p and miR-375 have been shown to promote resistance. These miRNAs are known to target PH domain and leucine-rich repeat protein phosphatase 1 (*PHLPP1*), a tumor suppressor gene that negatively regulates the AKT pathway. The sensitivity to cetuximab increases when miR-199-5p and miR-375 are suppressed [95].

According to a recent study, lncRNA MIR570HG level was found to be high in CRC cells resistant to regorafenib. This lncRNA acts as a sponge for miR-145 and inversely regulates the expression of miR-145. At this time, the expression of Smad3, a target of miR-145, increases, thereby promoting resistance to regorafenib [96]. The BRAF inhibitor vemurafenib is less effective in *BRAF*-mutated CRC. According to a recent study, miR-145 is significantly downregulated in vemurafenib-resistant cell lines. Therefore, it is important to increase the expression of miR-145 to enhance the sensitivity of cells to vemurafenib [97]. YAP is often overexpressed in patients with CRC with a low survival rate. Recent studies have reported that miR-550a-3-5p directly targets YAP to increase the sensitivity towards vemurafenib when resistance to the latter develops. This is also associated with a negative correlation between YAP and miR-550a-3-5p expression in CRC patients with low survival rates [98].

**Table 2:** MicroRNAs (miRNAs) involved in targeted drug resistance in colorectal cancer (CRC).

miRNA	Drug	Target Gene	Signaling Pathway	Level	Reference
miR-100	Cetuximab	DKK1, DKK3, ZNRF3, RNF43, APC2	Wnt/β-catenin	Up	[93]
miR-125b	Cetuximab	DKK1, DKK3, ZNRF3, RNF43, APC2	Wnt/β-catenin	Up	[93]
miR-302a	Cetuximab	NFIB, CD44	MAPK/AKT	Down	[94]
miR-199-5p	Cetuximab	PHLPP1	PI3K/AKT	Up	[95]
miR-375	Cetuximab	PHLPP1	PI3K/AKT	Up	[95]
miR-145	Regorafenib	SMAD3	TGF-β/SMAD	Down	[96]
miR-145	Vemurafenib	SMAD3	TGF-β/SMAD	Down	[97]
miR-550a-3-5p	Vemurafenib	YAP1	Hippo	Down	[98]

### 3.5. Roles of miRNAs in Immunotherapy

Unlike the direct targeting of cancer cells, immunotherapy promotes the activity of immune cells and modulates immune check points or the tumor microenvironment. Several previous studies have demonstrated immunotherapy-associated miRNAs that can regulate immune checkpoint molecules in natural killer cells, T helper cells, and cytotoxic T cells. Only one study on the regulation of PD-L1 expression by miR-148a-3p has been conducted in CRC. MiR-148a-3p inhibits PD-L1 expression and reduces T-cell apoptosis [99]. Although there is only one report on immunotherapy-related miRNA in CRC, we predict that immunotherapy-related miRNAs can prevent resistance in other cancer types.

MiR-5119 inhibits PD-L1 in the dendritic cells of breast cancer. Overexpressed miR-5119 reduces T-cell exhaustion and suppresses tumor growth in mouse models [100]. In lung cancer, miR-200 suppresses PD-L1, leading to the activation of CD8^+^ T cells and inhibition of metastasis [101]. The miR-23a/27a/24-2 cluster regulates M2 macrophage polarization in breast cancer. Ma et al. have shown that the overexpressed miR-23a/27a/24-2 cluster significantly decreases breast cancer progression owing to the targeting of JAK1/STAT6 by miR-23a and of IRF4/PPAR-𝛾 by mi-R27a [102]. MiR-142-3p also modulates M2 macrophages via the TGF-β signaling pathway [103]. MiR-23 contributes to immunity in different ways. It targets BLIMP-1, a transcription factor, and promotes the cytotoxicity of CD8^+^ cytotoxic T lymphocytes in lung cancer [104]. Wei et al. have shown that miR-138 can also be a candidate for immunotherapy as it inhibits PD-1 and T-lymphocyte-associated molecule 4, activating antitumor immune effects in glioblastoma [105].

## 4. Clinical Application of miRNAs as Anti-Cancer Drug Resistance Biomarkers and Therapeutic Targets

**Figure 2:**
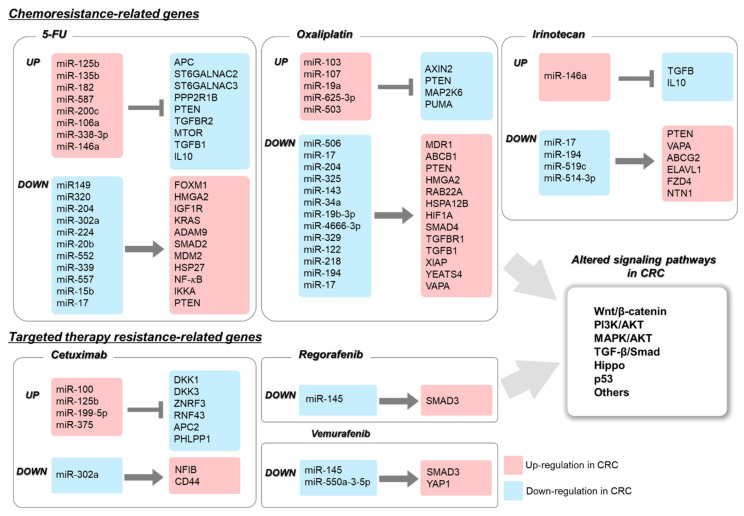
Summary for drug resistance-related miRNAs in CRC.

As describe above, miRNAs and its target genes, are strongly involved in anti-cancer drug resistance via abnormal expression of oncomiRNAs and/or tumor suppressor miRNAs in CRC (Figure 2). Increasing evidence indicates that miRNA levels can be used as biomarkers. Recent studies have shown that circulating miRNAs, including exosomal and naked miRNAs in liquid biopsies of blood, urine, and milk can be used as non-invasive prediction markers of drug response. Tumor-derived exosomes (50–100 nm) were first investigated in the peripheral circulation of patients with cancer [106,107]. The presence of certain miRNAs in the plasma or ascitic fluids of patients with cancer have led researchers to investigate the role of exosomal miRNAs as biomarkers in disease diagnosis [108]. In particular, the exosome-derived miRNAs stably circulate in body fluids in a cell-free form and might reflect the miRNA signature of the parental or metastatic tumor [109]. Thus, secreted exosomes are associated with disease status, such as stage and tumor progression as well as drug resistance [88,110,111,112]. Therefore, exosomal miRNAs are useful for predicting both strategy of chemotherapy as well as drug resistance.

The ability of miRNAs to control cellular processes by regulating various targets underscores their potential as a therapeutic tool for cancer treatment. Currently, two theories have been proposed for treating patients with cancer using miRNA-based therapy. The first is the miRNA inhibition therapy. OncomiRs are frequently overexpressed in human cancers, including in CRC, and their inhibition can reduce oncogenic functions by increasing the expression of target tumor suppressor genes. Inhibitors targeting specific miRNAs comprise complementary single-stranded oligonucleotides, the mature forms of which cannot be processed by RISC. Currently, several types of inhibitors, including anti-miR oligonucleotide, locked nucleic acid, miRNA sponges, and small molecules are being used [113,114,115,116,117,118,119,120]. The miRNA inhibition therapy can be applied to target the drug resistance-related miRNAs. MiR-125b and miR-146a are overexpressed in chemoresistant CRC patients; therefore, suppression of these miRNAs via miRNA inhibitors may restore the normal expression and functions of their targets, which are associated with drug-resistant genes. The second is the miRNA mimetic agent therapy [121,122]. MiRNA mimics, such as chemically modified RNA duplexes, increase the target miRNA expression artificially, resulting in restoration of expression and functions of tumor suppressive miRNAs [123,124,125,126]. MiRNA mimics in RISC inhibit downstream target mRNAs. Accumulating evidence shows the efficiency of miRNA mimic therapy in in vitro and in vivo models. For instance, the miR-143/145 cluster is a tumor suppressor that is frequently downregulated in several tumors, including in CRC. MiR-143/145 directly targets K-RAS and insulin-receptor substrate 1 [127,128]. Therefore, ectopic expression of the miR-143/145 cluster after transfection of its mimics decreased migration and invasiveness of CRC cells. MiR-192 is known as a tumor suppressor gene, as it directly inhibits the Ras-related protein Rab-2A in CRC [129]. MiR-192 was downregulated in CRC; therefore, introduction of miR-192 can be used as a therapeutic strategy for treating CRC. Wu et al. attempted to modify the miR-129 mimic by replacing uracil with 5-FU to generate miR-129 linked with 5-FU (Mimic-1) [130]. The therapeutic effects of Mimic-1 were confirmed in vitro and in vivo and were evident from the suppression of cell proliferation and CRC metastasis. Therefore, the miRNA mimic therapy might be a novel therapeutic approach for treatment of many cancers, including those showing drug resistance.

Currently, several groups are attempting to combine chemotherapy or targeted therapy with RNA interference to downregulate drug resistance-related genes. Using miRNA mimetic agents or miRNA inhibitors, resistant cells can be transiently sensitized to anti-cancer drugs owing to the modulation of miRNA target genes. Subsequently, treatment with therapeutic agents might be useful for treating the drug-resistant cancer. MiRNAs can suppress drug resistance-related pathways, which include genes involved in DNA repair, cell cycle, and apoptosis; therefore, miRNAs can sensitize drug-resistant tumor cells. For example, miR-21 has been shown to negatively regulate the core mismatch repair recognition protein complex human mutS homolog 2 (hMSH2) and 6 (hMSH6). Therefore, miR-21 expression reduces 5-FU-induced G2/M damage arrest and apoptosis, suggesting that its downregulation could improve therapeutic efficacy in CRC [131]. Another report showed that miR-129 promotes apoptosis by inhibiting *BCL2* and enhancing the cytotoxic effect of 5-FU in CRC cells [132]. In addition, the identification of regulatory miRNAs for multidrug resistance (MDR) transporters including ATP-binding cassette (ABC) transporters is important to improve sensitivity to anti-cancer drugs by suppressing the export of drugs. For example, the downregulation of miR-26b decreases the sensitivity of CRC cells to 5-FU, because of an increase in the expression of its target gene, P-glycoprotein (P-gp) [133]. A recent report suggests that the ectopic expression of miR-298 reduces P-gp expression, thereby increasing the cellular accumulation of antiepileptic drugs (AEDs) in drug-resistant glioblastoma cells [134]. The other MDR gene, ABCB1 is targeted by miR-4539, resulting in enhanced sensitivity to doxorubicin in triple negative breast cancer cells [135]. Therefore, targeting of miRNAs associated with chemotherapy or targeted therapy resistance might enhance clinical outcomes with general tumor therapy. Furthermore, it is possible that the co-administration of standard therapeutics with miRNA drugs could assist in overcoming anti-cancer drug resistance in CRC.

Although chemically modified miRNAs are more stable than the unmodified miRNAs, they cannot be applied in vivo because of low efficacy of delivery. Researchers have attempted to improve effective miRNA delivery using nanoparticles, viral vectors, lipid-based nanocarriers, polymeric vectors, dendrimer-based vectors, and cell-derived membrane vesicles [136,137,138,139,140,141,142]. To stably deliver the miRNAs in the target tissues, the delivery systems must overcome the major hurdles of high efficiency and low toxicity. For example, exosome-transmitted miRNAs have been shown to increase chemosensitivity of oxaliplatin-resistant CRC [143]. Liu et al. found that miR-128-3p-transfected fetal human cell line effectively packaged miR-128-3p into secreted exosomes. Introduction of the miR-128-3p packaged exosomes into oxaliplatin-resistant cells improved oxaliplatin response in CRC cells both in vitro and in vivo. Therefore, exosomes or any other effective miRNA delivery system may enhance the chances of increasing the chemosensitivity of CRC cells.

## 5. Conclusions

Drug resistance is considered a major obstacle in treating cancer. Several previous studies that have attempted to uncover the mechanisms of drug resistance have developed novel therapeutic approaches. In this review, we described miRNAs as diagnostic markers or therapeutic targets for treating drug resistance in CRC. We highlighted examples of miRNAs that participate in the gene expression regulatory network and discussed how they contribute to anti-cancer drug resistance. Although many studies have used miRNAs to predict drug response in CRC, the lack of a complete understanding regarding the mechanisms via which miRNAs regulate drug resistance has remained a major limitation. As miRNAs and their target mRNAs can be potential therapeutic targets and diagnostic markers for drug resistance in patients with CRC, further studies are necessary to identify key miRNAs and their targets and to elucidate the functions and mechanisms of drug resistance-related miRNAs.

## Data Availability

The data presented in this study are available on request from the corresponding author.
